# Supplementary material for: Consumption of Galactose by Trypanosoma cruzi Epimastigotes Generates Resistance against Oxidative Stress
Source: Pathogens. 2022 Oct 11;11(10):1174. doi: 10.3390/pathogens11101174 (PMC9611177; doi:10.3390/pathogens11101174)
Supplement: Supplementary file 1 [file pathogens-11-01174-s001.zip › pathogens-1900985-supplementary.pdf]

Consumption of galactose by *Trypanosoma cruzi* epimastigotes generates resistance against oxidative stress.

Ángel Lobo-Rojas*, Ender Quintero-Troconis*, Rocío Rondón-Mercado, Mary Carmen Pérez-Aguilar., Juan Luis Concepción and Ana Judith Cáceres.Laboratorio de Enzimología de Parásitos, Departamento de Biología, Facultad de Ciencias, Universidad de Los Andes, Mérida, Venezuela.

* Corresponding Authors:

aeloborojas@gmail.com, ejqtroconis@gmail.com

Supplementary material

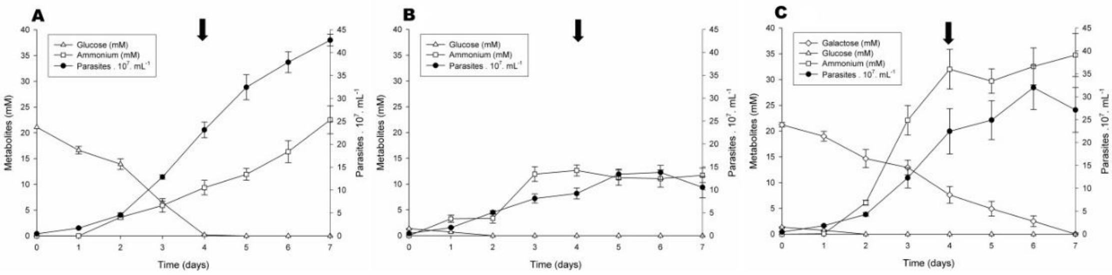

Figure S1. Growth of *Trypanosoma cruzi* epimastigotes in presence of different carbon sources. All cultures of epimastigotes (100 mL) were started with 5 × 10^6 cells/mL and incubated at 28 °C and constant shaking (160 RPM). Growth was followed by counting number of parasites in a Neubauer chamber; samples were collected every 24 hours until the culture reached stationary phase. Supernatants of media were used to determine glucose, galactose and ammonium concentrations. (**A**) shows the growth profile of parasites in LIT-medium supplemented with glucose 20 mM, (**B**) shows the growth profile of parasites in LIT-medium without supplementing with any hexose (glucose 1.5 mM) and (**C**) shows the growth profile of parasites in LIT-medium supplemented with galactose 20 mM. Black arrows denote the time of parasite collection for oxidative stress experiments and specific activities measurements.

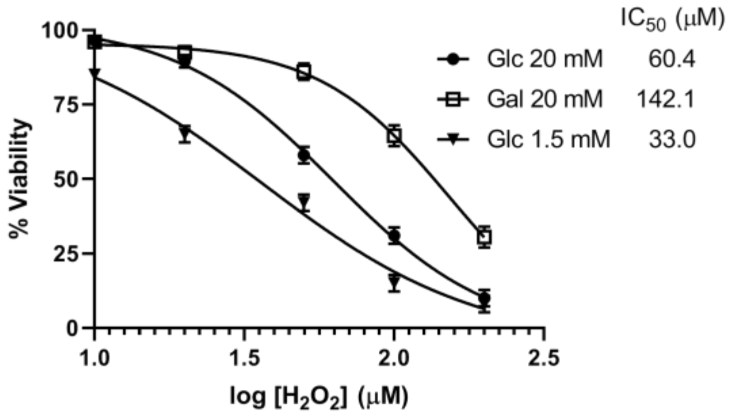

Figure S2. Effect of carbon and energy source on the IC_{50} showed for *Trypanosoma cruzi* epimastigotes against hydrogen peroxide. Epimastigotes were grown on the respective primary carbon source as described on section *4.1,* washed three times with the respective OSB buffer and the experiment started with the addition of different concentrations of hydrogen peroxide (0 – 200 μM), after two hours of incubation with shaking of 140 RPM, parasites were washed three times and counted viable and death cells in a Neubauer chamber by using the Trypan blue exclusion test (see section *4.4*). Data showed was obtained as a mean of three independent experiments. Curves and IC_{50} values were obtained by analyzing the data with GraphPad Prism 8 software.

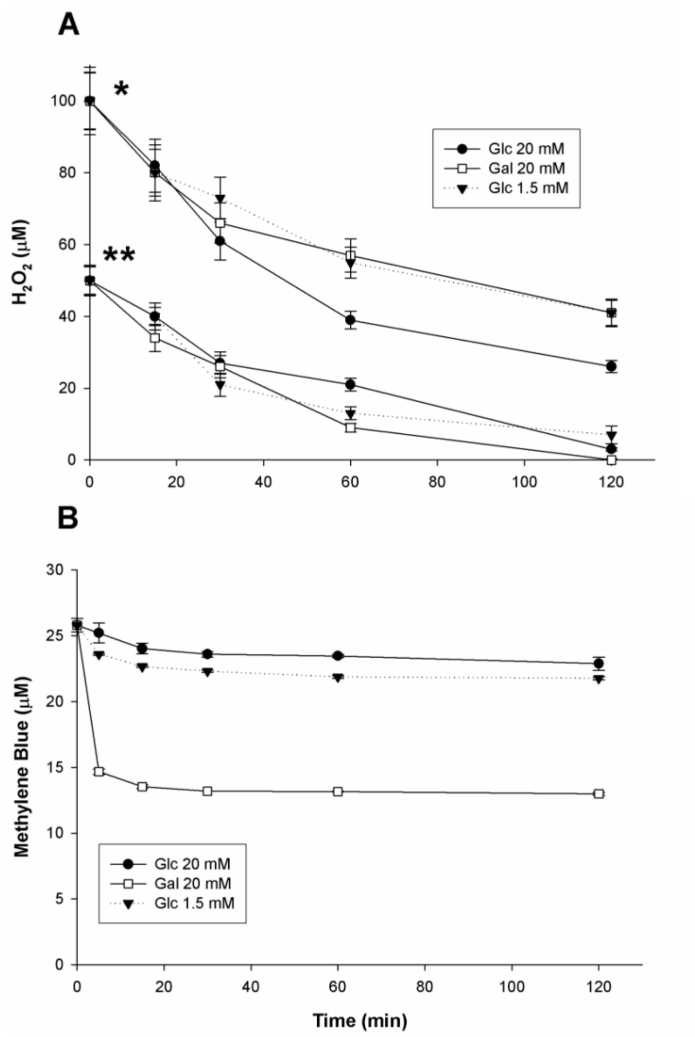

Figure S3. Concentration of hydrogen peroxide (A) and methylene blue (B) during the experiment of oxidative stress evaluation of epimastigotes of *T. cruzi* (see sections *2.1* and *2.2*). (**A**) The concentrations of hydrogen peroxide were quantified at different times (0, 15, 30, 60 and 120 minutes), following the protocol described in section *4.5*. Single asterisk represents the condition of hydrogen peroxide 100 µM and double asterisk the condition of hydrogen peroxide 50 µM. (**B**) Concentrations of methylene blue was quantified at different times (0, 5, 15, 30, 60 and 120 minutes), following the protocol described in section *4.5*.

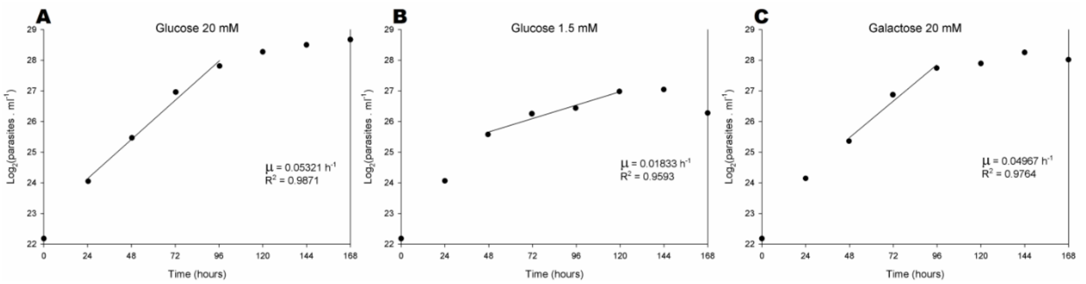

Figure S4. Linearized growth profile of *Trypanosoma cruzi* epimastigotes in presence of different carbon sources. The growth rate constant (μ) in exponential phase of each growth condition was calculated as the slope of the log2 of concentration of parasites per milliliter versus time. (**A**) corresponding to growth rate of epimastigotes in presence of glucose 20 mM, μ was calculated by estimating the slope in exponential phase between 24 and 96 hours, while in (**B**) and (**C**), corresponding to growth rates of epimastigotes in presence of glucose 1.5 mM and galactose 20 mM respectively, μ was calculated as the slope in exponential phase after 48 hours which is the time when entire glucose is exhausted in the media of both conditions (see supplementary figure 2).

Table S1. Statistical analysis of the experiment shown in figure 1. P-values were calculated in every time interval and every concentration tested, using a two-tailed, unpaired samples t-test, calculated with GraphPad Prism 8 software. Gal: Condition in experiments with 20 mM galactose, Glc: Condition in experiments with 20 mM glucose, LGlc: Condition in experiments with 1.5 mM glucose. For more information see section 4.3.

**Hydrogen peroxide**	**0 µM**					**50 µM**					**100 µM**				
Conditions/Time(min)	0	15	30	60	120	0	15	30	60	120	0	15	30	60	120
Gal-Glc	1	0.5734	0.5734	0.2879	1	1	0.0009	0.001	0.0006	0.0012	1	0.0002	0.0007	0.0006	0.0007
Gal-LGlc	1	0.1249	0.0535	0.0027	0.0022	1	0.0005	0.0002	0.0003	0.0003	1	0.0003	0.0006	0.0004	0.0001

Glc-LGlc	1	0.0808	0.0363	0.0044	0.0022	1	0.0056	0.0061	0.0734	0.0097	1	0.5838	0.1252	0.0726	0.0148

Table S2. Statistical analysis of the experiment shown in figure 2. P-values were calculated in every time interval and every concentration tested, using a two-tailed, unpaired samples t-test, calculated with GraphPad Prism 8 software. Gal: Condition in experiments with 20 mM galactose, Glc: Condition in experiments with 20 mM glucose, LGlc: Condition in experiments with 1.5 mM glucose. For more information see section 4.3.

**Methylene blue**	**0 µM**					**30 µM**					
Conditions/ Time(min)	0	15	30	60	120	0	5	15	30	60	120
Gal-Glc	1	0.5734	0.5734	0.2879	1	1	0.4442	0.2562	0.0001	0.0002	0.0002
Gal-LGlc	1	0.1249	0.0535	0.0027	0.0022	1	0.0906	0.0121	0.0128	0.0013	0.0001
Glc-LGlc	1	0.0808	0.0363	0.0044	0.0022	1	0.042	0.0136	0.2862	0.9809	0.4062
